# A healing challenge: examining NHS staff sickness absence rates

**DOI:** 10.1177/01410768231223779

**Published:** 2024-02-12

**Authors:** Mohammed Blaaza, Lara Shemtob, Kaveh Asanati, Azeem Majeed

**Affiliations:** School of Public Health, Imperial College London, St Mary's Hospital, London W2 1PG, UK

Effective healthcare delivery within the National Health Service (NHS) in England relies on maintaining adequate staffing levels across all professional groups. Insufficient medical and nursing coverage is associated with risks to patient outcomes.^1^ Sickness absence within the NHS can impact services, patient care and costs,^2^ and has an impact on workforce retention, which feeds back into staffing shortages.^3^ Exploring the data provided by NHS Digital on sickness absence rates among NHS staff reveals trends that may have implications for workforce well-being and healthcare delivery.

## Overall sickness absence rates

Data published by NHS Digital reveal a steady increase in sickness absence rates across all staff groups within the NHS since 2009. Sickness absence rates surged during the coronavirus disease 2019 (COVID-19) pandemic from 2020 onwards and have remained elevated compared to pre-pandemic levels.

During the 2010s, monthly sickness absence rates for NHS healthcare professionals generally fluctuated between 4% and 5% with expected seasonal variation. During the pandemic, the fluctuations in absence rates became more pronounced, with peaks at 6%. The majority of COVID-19 restrictions had been lifted by January 2022, but since then, NHS sickness absence rates have remained elevated compared to the period preceding the pandemic, between 5% and 6% with smaller degrees of seasonal fluctuations. The most recent data from May 2023 suggest that the rates appear to be trending downwards towards pre-COVID-19 levels. The data generally demonstrate seasonal variation with peaks during the winter months, correlated with rise in seasonal illnesses such as respiratory infections.

Data from the Office for National Statistics show that sickness absence in the UK labour force generally remained stable at around 2% before the pandemic.^4^ Since 2020, the sickness absence rate has been steadily rising to a peak of 2.6% in 2022. NHS employees tend to have around double the rates of sickness absence of the UK labour force as a whole.

## Reasons for sickness absence

Mental ill health, particularly anxiety and depression, was consistently the most common reason for sickness absence within the NHS, including during the pandemic. The data for all people in employment show that minor illnesses rather than mental illness account for the highest percentage of time lost. Mental illness accounts for 8%–12% of sickness absence each year in the UK labour force, compared with around 25% per year in the NHS workforce. Hence, NHS employees are two to three times more likely to require sickness absence leave for mental ill health than the average UK employee. This likely relates to working conditions in the NHS, with over one-third of staff surveyed in the 2022 NHS staff survey in England reporting they often or always felt burnt out because of their work.^5^ High-stress environments, common within the NHS, can worsen anxiety and exacerbate mental ill health.^6^ Shift work among healthcare professionals can have negative systemic effects.^7^ The physical demands of certain roles may precipitate and exacerbate musculoskeletal injuries.

## Sickness absence rates by staff group

Sickness absence rates vary among different healthcare professional groups. Doctors of all grades generally have significantly lower sickness absence rates compared with other healthcare staff, generally below 2%. Nursing staff, ambulance staff and allied health professionals had much higher rates. Reasons for this discrepancy could be because some staff groups are less likely to record sickness absence, or may have role flexibility that allows them to work remotely when not well enough to undertake face-to-face work. It is also possible that some staff groups experience more ill health, affecting their ability to work due to working conditions, or that others have a higher threshold to take sickness absence when unwell, leading to presenteeism, which is also unlikely to be good for either them or the organisation. I can confirm we are happy with this change.

## Implications for policy and practice

The NHS workforce plan must address sickness absence rates across healthcare services, but falls short of a data-driven strategy.^8^ The plan looks to the national Growing Occupational Health and Wellbeing strategy for a way forward. However, given that access to occupational health services is already better within the NHS than most other parts of the economy, this is unlikely to be a complete solution. Access to occupational health and specialist services, including stress management courses, and improving staff access to mental health treatment and resources will help but are primarily a reactive approach. Instead, NHS organisations need to work with occupational health leaders to take proactive measures to understand the problems in working conditions, assess risk and implement change higher up the hierarchy of controls where necessary. This may require addressing system factors such as demand outweighing resource that involve decisions higher up the policy agenda than any one NHS organisation can take.

There is a need to better understand the reasons behind elevated rates of sickness absence in the NHS workforce compared with the average in the UK workforce to effectively close this gap. This would involve understanding the reasons behind high rates of sickness absence due to mental ill-health and the varying rates of sickness absence in different professional groups. Comparison to sickness absence rates across the public sector rather than the economy as a whole shows less of a gap but does not eliminate the higher sickness absence rates in the NHS.^9^ Comparing sickness absence, working conditions and access to health and work support in the NHS to other healthcare workforces would be informative, as would be looking for differences between individual NHS Trusts to understand what is working well to keep sickness absence rates lower. The national data suggest that working conditions have a significant role to play in disproportionately high sickness absence rates in the NHS workforce. Ultimately, this is what needs to be addressed for any improvements to be sustainable.

## Declarations

### Guarantor

MB, LS, KA and AM.

### Provenance

Not commissioned; editorial review.
